# 5-Fluorouracil blocks quorum-sensing of biofilm-embedded methicillin-resistant *Staphylococcus aureus* in mice

**DOI:** 10.1093/nar/gkab251

**Published:** 2021-04-15

**Authors:** Ferdinand Sedlmayer, Anne-Kathrin Woischnig, Vincent Unterreiner, Florian Fuchs, Daniel Baeschlin, Nina Khanna, Martin Fussenegger

**Affiliations:** Department of Biosystems Science and Engineering, ETH Zürich, Mattenstrasse 26, CH-4058 Basel, Switzerland; Laboratory of Infection Biology, Department of Biomedicine, University and University Hospital Basel, Hebelstrasse 20, CH-4031 Basel, Switzerland; Novartis Institutes for BioMedical Research (NIBR), Chemical Biology and Therapeutics (CBT), CH-4033, Basel, Switzerland; Novartis Institutes for BioMedical Research (NIBR), Chemical Biology and Therapeutics (CBT), CH-4033, Basel, Switzerland; Novartis Institutes for BioMedical Research (NIBR), Chemical Biology and Therapeutics (CBT), CH-4033, Basel, Switzerland; Laboratory of Infection Biology, Department of Biomedicine, University and University Hospital Basel, Hebelstrasse 20, CH-4031 Basel, Switzerland; Division of Infectious Diseases and Hospital Epidemiology, University Hospital of Basel, Petersgraben 4, CH-4031 Basel, Switzerland; Department of Biosystems Science and Engineering, ETH Zürich, Mattenstrasse 26, CH-4058 Basel, Switzerland; Faculty of Science, University of Basel, Mattenstrasse 26, CH-4058 Basel, Switzerland

## Abstract

Antibiotic-resistant pathogens often escape antimicrobial treatment by forming protective biofilms in response to quorum-sensing communication via diffusible autoinducers. Biofilm formation by the nosocomial pathogen methicillin-resistant *Staphylococcus aureus* (MRSA) is triggered by the quorum-sensor autoinducer-2 (AI-2), whose biosynthesis is mediated by methylthioadenosine/*S*-adenosylhomocysteine nucleosidase (MTAN) and *S*-ribosylhomocysteine lyase (LuxS). Here, we present a high-throughput screening platform for small-molecular inhibitors of either enzyme. This platform employs a cell-based assay to report non-toxic, bioavailable and cell-penetrating inhibitors of AI-2 production, utilizing engineered human cells programmed to constitutively secrete AI-2 by tapping into the endogenous methylation cycle via ectopic expression of codon-optimized MTAN and LuxS. Screening of a library of over 5000 commercial compounds yielded 66 hits, including the FDA-licensed cytostatic anti-cancer drug 5-fluorouracil (5-FU). Secondary screening and validation studies showed that 5-FU is a potent quorum-quencher, inhibiting AI-2 production and release by MRSA, *Staphylococcus epidermidis, Escherichia coli* and *Vibrio harveyi*. 5-FU efficiently reduced adherence and blocked biofilm formation of MRSA in vitro at an order-of-magnitude-lower concentration than that clinically relevant for anti-cancer therapy. Furthermore, 5-FU reestablished antibiotic susceptibility and enabled daptomycin-mediated prevention and clearance of MRSA infection in a mouse model of human implant-associated infection.

## INTRODUCTION

Molecular communication among bacteria by means of small diffusible signaling molecules, known as quorum sensing, serves to coordinate inter- and intra-population behavior (1). The most common quorum-sensing communication signal is autoinducer-2 (AI-2) (2,3), which controls virulence and biofilm formation in various human pathogens, such as methicillin-resistant *Staphylococcus aureus* (MRSA), thereby contributing to their antibiotic resistance (4). In the industrialized world, the rise of antibiotic-resistant nosocomial infections has reached crisis proportions; in particular, implant-associated MRSA infections (5) account for almost 50% of infections that occur following prosthetic surgery, and are associated with dramatic morbidity and exploding healthcare expenditures (6,7). This situation calls for immediate (8) and concerted action to develop alternative treatment options that can replace or complement antibiotics (9). Molecular interference with the pathogens’ quorum sensing, i.e., quorum quenching (10), by using degradative enzymes (11) or small-molecular inhibitors to block the synthesis and release of quorum-sensing signaling compounds, is one possible approach to switch off biofilm formation (12,13) and reduce or eliminate antibiotic resistance (14). In this context, AI-2 is synthesized in two sequential steps: 5′-methylthioadenosine nucleosidase (MTAN)-catalyzed hydrolysis of cellular S-adenosylmethionine (SAM) to afford S-ribosylhomocysteine (SRH), followed by *S*-ribosylhomocysteine lyase (LuxS)-mediated conversion of SRH to AI-2 (15). Thus, inhibition of MTAN and/or LuxS is expected to quench the quorum-sensing capability of targeted pathogens and consequently to attenuate their virulence and antibiotic resistance.

Screening for enzyme-inhibiting compounds is standard practice in drug discovery and development, and typically involves microscale activity assays (16), combinatorial shuffling in nanocompartments (17) and virtual screening methods (18). However, most of these in-vitro strategies provide little or no information on the function, toxicity, bioactivity and bioavailability of the hit compounds and most hits will fail during later stages of drug development (19). On the other hand, human cell-based assays may provide an all-in-one opportunity to detect bioavailable, cell-permeable, non-cytotoxic and target-specific functional drug candidates (20–22). Prime examples would be the anti-tuberculosis drugs that are currently being validated by a Bioversys-GlaxoSmithKline consortium in phase-I clinical trials (23).

5-Fluorouracil (5-FU) was licensed in 1962 for the treatment of various common and aggressive cancers, including colon cancer, breast cancer and pancreatic cancer, and is on the World Health Organization's List of Essential Medicines, which defines the most effective and safe medicines needed in a public health system (https://www.who.int/medicines/publications/essentialmedicines/en/). 5-FU principally acts as a thymidylate synthase inhibitor, blocking the synthesis of thymidine monophosphate (dTMP), which is a nucleoside required for DNA replication (24). Administration of 5-FU triggers apoptosis of rapidly dividing cancer cells by depriving them of dTMP.

Here, we describe the development and application of a high-throughput screening platform for inhibitors of MTAN and/or LuxS, built on a cell-based assay employing engineered human cells programmed to constitutively secrete AI-2. Notably, we found that one of the hit compounds, 5-FU, blocks quorum-sensing by MRSA and prevents biofilm formation at an order-of-magnitude-lower concentration than that clinically relevant for anti-cancer therapy. Furthermore, 5-FU enables daptomycin-mediated prevention and clearance of MRSA infection in a mouse model of human implant-associated infections. We believe this finding has the potential to be rapidly translated into clinical use.

## MATERIALS AND METHODS

### Microbial strains

*Escherichia coli* strains XL10-Gold^®^ (XL10-Gold^®^ ultracompetent cells, Agilent Technologies, Basel, Switzerland; cat. no. 200314) and RP437 (CGSC#: 12122; Coli Genetic Stock Center, Yale, USA) were used for AI-2 accumulation experiments. *Escherichia coli* was grown at 37°C on LB agar plates or in liquid LB medium (Beckton Dickinson, NJ, USA; cat. no. 244610) supplemented with appropriate antibiotics (ampicillin, 100 μg/ml, cat. no. A9518; kanamycin, 30 μg/ml, cat. no. K1377; both from Sigma-Aldrich, Munich, Germany). To detect AI-2, the *Vibrio harveyi* strain MM32 (luxN::Cm, luxS::Tn5Kan, ATCC: BAA-1121) was propagated at 30°C in AB-Medium (0.3 M NaCl, 10 mM potassium phosphate, 1 mM l-arginine, 10 mM potassium phosphate, 0.1 mM arginine, 1% glycerol) supplemented with kanamycin (30 μg/ml). *Staphylococcus epidermidis* (SE) 1457 wild type (wt) and methicillin-resistant *S. aureus* (MRSA) ATCC 43300 were used for the in vitro AI-2 inhibition and MRSA for the *in vivo* experiments. Both staphylococcal strains were grown in Tryptic Soy Broth (TSB) (Becton Dickinson AG, Allschwil, Switzerland) overnight without shaking at 37°C. For the *in vitro* experiments the overnight SE culture was diluted to 1 – 3 × 10^7^ CFU/ml and the overnight MRSA culture to 2 – 3 × 10^5^ CFU/ml. For the in vivo experiments, MRSA 43300 was washed twice with saline (Bichsel AG, Interlaken, Switzerland) and diluted to the appropriate bacteria load.

### Cell culture, transfection and stable cell line generation

Human embryonic kidney cells (HEK-293T, ATCC: CRL-11268) were cultivated in Dulbecco's modified Eagle's medium (DMEM; Thermo Fisher, Basel, Switzerland; cat. no. 31966–021) supplemented with 10% (v/v) fetal calf serum (FCS; Bioconcept, Allschwil, Switzerland; cat. no. 2-01F10-I; lot no. D10839D) and 1% (v/v) penicillin/streptomycin solution. All cells were cultured at 37°C in a humidified atmosphere containing 5% CO_2_.

LuxS (MF688636) and MTAN (MF688635) transgenes derived from *E. coli* were codon-optimized for stable expression in mammalian cells. For the generation of double stable HEK-293-derived cell line HEK-293_AI2_ transgenic for simultaneous constitutive P_hEFIα_-driven MTAN and P_hCMV_-driven LuxS-eYFP expression, 250,000 cells were first transfected with 2000 ng of pFS168 (P_hEFIα_-MTAN-pA) and selected in culture medium containing 20 μg/ml blasticidin (cat. no. ant-bl-1; InvivoGen, San Diego, CA, USA) for two weeks. Following expansion of single clones by limiting dilution for another two weeks and validation of functional MTAN expression, the best-performing clone (HEK-293_sFS25c21_) was co-transfected with 1660 ng of fluorescent *S*-ribosylhomocysteinase (LuxS-eYFP; P_hCMV_-luxS-eYFP-pA, pFS169) and 340 ng of the zeocin resistance encoding plasmid pZeoSV2(+). After 17 days of selection in medium containing 200 μg/ml (w/v) zeocin (cat. no. ant-zn-1; Invivogen, San Diego, CA, USA) and 20 μg/ml blasticidin, resistant monoclonal cells (HEK-293_sFS26cx_) were obtained by limiting dilution cloning and screened for AI-2 activity in their supernatants. The best performing clone 12 was named HEK-293_AI2_. The cell line was regularly tested for the absence of mycoplasma.

### Compound screening

20 μl of HEK-293_AI2_ cells (10^4^ cells per well) were resuspended in 20 μl Dulbecco's modified Eagle's medium (DMEM; Thermo Fisher, Basel, Switzerland, low glucose, without phenol red, cat. no. 11880–028) supplemented with 4 mM l-glutamine (Thermo Fisher, Basel, Switzerland, cat. no. 25030-024), 30 μg/ml kanamycin and 1% FCS and seeded into a poly-l-lysine-coated Black Greiner clear-bottom 384-well plate (cat. no. 3845; Corning, New York, USA) using a Multidrop Combi Reagent Dispenser (cat. no. 5840300; Thermo Fisher, Basel, Switzerland). Cells were incubated for 20 h and then 20 nL of individual compounds (stock solution in 5 mM in DMSO) or controls (stock solutions of novobiocin, 10 mM in DMSO; staurosporin, 20 mM in DMSO) were transferred with Echo 550 liquid handler (Labcyte, San Jose, CA, USA) into the plate.

### AI-2 quantification for screening

Compound-treated cells were cultivated for 24 hours and the cell culture supernatants (10 μl) were transferred to dry black polystyrene 384-well plates (Corning, New York, USA, cat. no. 3571). The AI-2 activity was quantified by adding 40 μl per 384-well of *V. harveyi* MM32 AI-2 reporter strain, diluted 1:500 in AB-Medium from a stationary overnight culture grown in Luria Marine (LM)-medium containing 20 g of NaCl, 10 g of Bacto Tryptone (Difco Laboratories), and 5 g of yeast extract (BBL) (25). Plates were sealed with BREATHseal™ (cat. no. 7.676 050; Greiner Bio-One, Frickenhausen, Germany) and incubated for 4 h at 30°C and 200 rpm on a Multitron Pro shaker (Infors, Bottmingen, Switzerland). Bioluminescence was measured on an Envision 2104 Multilabel plate reader (Perkin Elmer, Waltham, USA) with 1 s integration time. For each plate, the luminescence data were normalized to the active controls (0% activity: Novobiocin; 100% activity: DMSO) measured in 8-fold replicates on each plate and expressed as % activity of DMSO control cells (100% activity). The initial hit inclusion criteria were a toxicity score of less than 50% and an AI-2 activity reduction below the average of all compound activity three times the standard deviation (-49.39% of activity).

### Z’ determination

The statistical effect size was calculated according to ${\boldsymbol Z}^{\prime} = {\boldsymbol 1} - ( {\frac{{{\boldsymbol 3}( {{{\boldsymbol \sigma} _{\boldsymbol p}} + {{\boldsymbol \sigma} _{\boldsymbol n}}} )}}{{( {{{\boldsymbol \mu} _{\boldsymbol p}} - {{\boldsymbol \mu} _{\boldsymbol n}}} )}}} )$, based on the equation described by Zhang *et al.* (1999) with the means (μ) and standard deviations (σ) of the luminescence resulting from positive control (p; novobiocin, 10 μM) and negative control (n; DMSO) supernatants.

### Resazurin-based viability assay

To monitor viable cells with active metabolism, we employed a fast resazurin assay. In short, 1 μl of PrestoBlue reagent (cat. no. A13261; Thermo Fisher, Basel, Switzerland) was added directly to each well of a 384-well plate by a Multidrop Combi Reagent Dispenser (cat. no. 5840300; Thermo Fisher, Basel, Switzerland). The plate was incubated for 1 h (37°C, 5% CO_2_), and then the fluorescence was measured with an Envision 2104 Multilabel plate reader (Perkin Elmer, Waltham, USA) at excitation and emission wavelengths of 560/9 nm and 590/20 nm, respectively. To calculate the relative cell viability, the fluorescence of DMSO-treated cells was set to 100%.

### Criteria for further evaluation of hits

Compounds for further evaluation were selected according to the following combined inclusion criteria: (i) lack of reported antibiotic activity according to PubChem, (ii) IC_50_ < 4 μM, (iii) lack of short-term cytotoxicity (viability decrease < 25%).

### AI-2 inhibition in *V. harveyi* BB170, *E. coli* RP437, SE 1457 wt, and MRSA ATCC 43300

The test strain *V. harveyi* BB170 was grown overnight in Luria Marine (LM)-medium (25). The culture was 1:500 diluted in AB-medium (0.3 M NaCl, 10 mM potassium phosphate, 1 mM l-arginine, 10 mM potassium phosphate, 0.1 mM arginine, 1% glycerol) the next day, and then challenged with various chemical compounds. After 5 h of shaking at 200 rpm at 30°C, the bioluminescence of BB170 was quantified on a GENios Pro plate reader (Tecan, Männedorf, Switzerland).

The test strain *E. coli* RP437 was grown overnight in LB. Its optical density was measured on a Novaspec II photometer (Pharmacia, Freiburg, Germany) and the culture was diluted to a final OD_600_ of 0.1 the next morning. The bacteria were challenged with test compounds for 3 h, and then AI-2 activity in the culture supernatant was determined. Specifically, 90 μl of the AI-2 reporter strain *V. harveyi* (MM32), grown overnight in LM medium and then prediluted 1:500 in AB-medium containing 30 μg/ml kanamycin, was added to 10 μl of *E. coli* supernatant (10% v/v) in black-bottomed Fluotrac 200 96-well plates (Greiner Bio-One, Frickenhausen, Germany), which were shaken at 200 rpm, at 30°C. The AI-2 induced bioluminescence of MM32 was measured after 5 h. Chemically synthesized DPD served (Omm Scientific, Dallas, Texas, USA) as a positive control.

The inhibition of AI-2 within the SE 1457 wt strain and the MRSA strain due to 5-FU (Sigma-Aldrich, Buchs, Switzerland) was measured after 3 h under static conditions at 37°C. To confirm that the phenotype could be restored, 1 μM synthetic (*S*)-4,5-dihydroxy-2,3-pentanedione (DPD) (Biosynth Carbosynth, Staad, Switzerland) was used. After 3 h under static conditions at 37°C, the supernatant was analysed for AI-2 activity with *V. harveyi* (MM32).

### Quantification of biofilm prevention

To quantify the formation of bacterial biofilms, bacteria were grown for 24 h in the presence of potential inhibitors in 96-well polystyrene plates. The plates were then washed three times with distilled water. Remaining cells were stained with 0.1% Crystal Violet solution (5% methanol, 5% isopropanol) and further washed to remove excess dye. Crystal Violet was redissolved in 20% acetic acid solution and the absorbance of the solution was measured at 600 nm.

To quantify the amount of adherent bacteria, 2 – 3 × 10^5^ CFU/ml MRSA were grown for 24 h in the presence of different 5-FU concentrations. After incubation under static conditions at 37°C, the plate was washed twice with PBS. Adherent bacteria were removed with 100 μl PBS and appropriate dilutions were plated on Mueller-Hinton agar (Becton Dickinson AG, Allschwil, Switzerland) plates overnight at 37°C. Three independent experiments were performed, each performed in triplicate, and the mean values were calculated. Dimethyl sulfoxide (DMSO) was used as a control.

### Evaluation of 5-FU for prophylaxis in a murine tissue cage infection model

To evaluate the prophylactic efficacy of 5-FU against MRSA infection, we used our murine model of foreign-body infection, which closely mimics human implant-associated infections. This model of foreign-body infection (26,27) was established with the approval of the Kantonale Veterinaeramt Basel-Stadt, Switzerland (permit no. 1710). Experiments were conducted according to the regulations of Swiss veterinary law and performed in the animal house of the Department of Biomedicine, University Hospital Basel, Switzerland. Healthy wild-type female C57BL/6 mice at 13 weeks of age (JanvierLabs, France) kept under specific pathogen-free conditions (biosafety level 2) were anesthetized, followed by the subcutaneous implantation of sterile cylindrical Teflon tissue cages (8.5 × 1 × 30 mm; volume: 1.9 ml) with 130 regularly spaced holes (Angst + Pfister AG, Zurich, Switzerland). They were housed in a 12 hr light/dark cycle (light from 7 am to 7 pm) in a temperature-controlled room (24°C) with free access to regular mice chow and water. Mice were randomly assigned to experimental groups, which were not involved in previous procedures. The mice were randomized into groups, which were treated as follows: saline (untreated growth control; *n* = 7), saline with 5.2844 mg/kg DPD (*n* = 4), 50 mg/kg daptomycin (DAP) (Novartis, Basel, Switzerland) (*n* = 11), 10 mg/kg 5-FU with and without 50 mg/kg DAP (each *n* = 8), 40 mg/kg 5-FU with or without 50 mg/kg DAP (*n* = 16 and *n* = 13, respectively), 40 mg/kg 5-FU with 1 μM DPD (*n* = 6), or 40 mg/kg 5-FU with 50 mg/kg DAP and 5.2844 mg/kg DPD (*n* = 8). DAP was given intraperitoneally immediately before implantation, and 5-FU and DPD was given immediately after implantation directly into the lumen of the cage. Afterwards, the cages were infected with 965 CFU of MRSA 43300. At 2 days post-infection, tissue cage fluid was collected, and the planktonic bacterial load was evaluated by plating on blood agar plates. In additional, mice were sacrificed and the tissue cages were explanted under aseptic conditions. The explanted tissue cages were washed twice with phosphate-buffered saline followed by 30 s vortexing, sonication for 3 min at 130 W and another 30 s vortexing to release adherent bacteria from the biofilm. Quantification of adherent bacteria was performed by plating appropriated dilutions on blood agar plates. To determine the prevention rate, the presence of any re-growth of MRSA was examined by further incubation of the cage in tryptic soy broth (TSB) for 48 h at 37°C. MRSA re-growth represents therapy failure, and the prevention rate was defined as the percentage of cages without growth in each treatment group.

### Statistical analysis

All in vitro data were analyzed with an unpaired parametric t test and expressed as mean and standard deviation (SD). All in vivo data were analyzed with the nonparametric Mann–Whitney *U* test because they did not show a normal distribution in the Shapiro–Wilk normality tests. Data are expressed as median and interquartile range (IQR). For all assays, a *P* value <0.05 was considered statistically significant. Statistical analysis was performed using Prism 9 (GraphPad Software, USA).

## RESULTS

### Design of the cell-based AI-2-specific quorum-quencher screening platform

**Figure 1.**
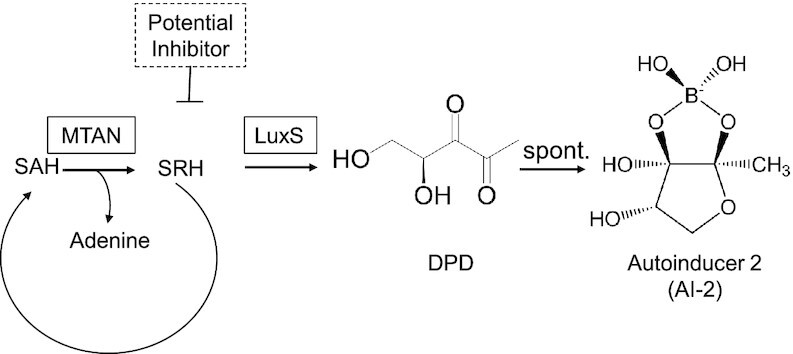
Schematic illustration of AI-2 biosynthesis. First, methylthioadenosine nucleosidase (MTAN) converts S-adenosylhomocysteine (SAH) into *S*-ribosylhomocysteine (SRH) as a part of the activated methyl cycle. SRH, in turn, serves as a substrate for S-ribosylhomocysteine lyase (LuxS), producing 4,5-dihydroxypentane-2,3-dione (DPD), which undergoes spontaneous rearrangements to form autoinducer 2 (AI-2).

Figure 1 shows a schematic illustration of the platform for screening bioavailable, non-cytotoxic and target-specific small-molecular drugs quenching the production of the biofilm- and virulence-promoting bacterial quorum-sensing molecule AI-2 (28,29). For the primary screen, we generated the double-transgenic human cell line HEK_AI-2_ constitutively co-expressing the codon-optimized bacterial AI-2-synthesizing enzymes 5′-methylthioadenosine nucleosidase (MTAN) and S-ribosylhomocysteine lyase (LuxS). In HEK_AI-2_, MTAN and LuxS tap into the activated methylation cycle of human cells to convert S-adenosylmethionine via S-ribosylhomocysteine into AI-2 (30). We previously showed in a proof-of-concept study (31) that the modular biosynthetic AI-2 production platform can function in HEK cells, and AI-2 production is significantly decreased by the addition of known MTAN or LuxS inhibitors such as immunicillin-A (32) and S-ribosylhomocysteine analogues (33). The decrease of AI-2 production induced by those inhibitors was not due to a decrease of cell viability (31).

**Figure 2.**
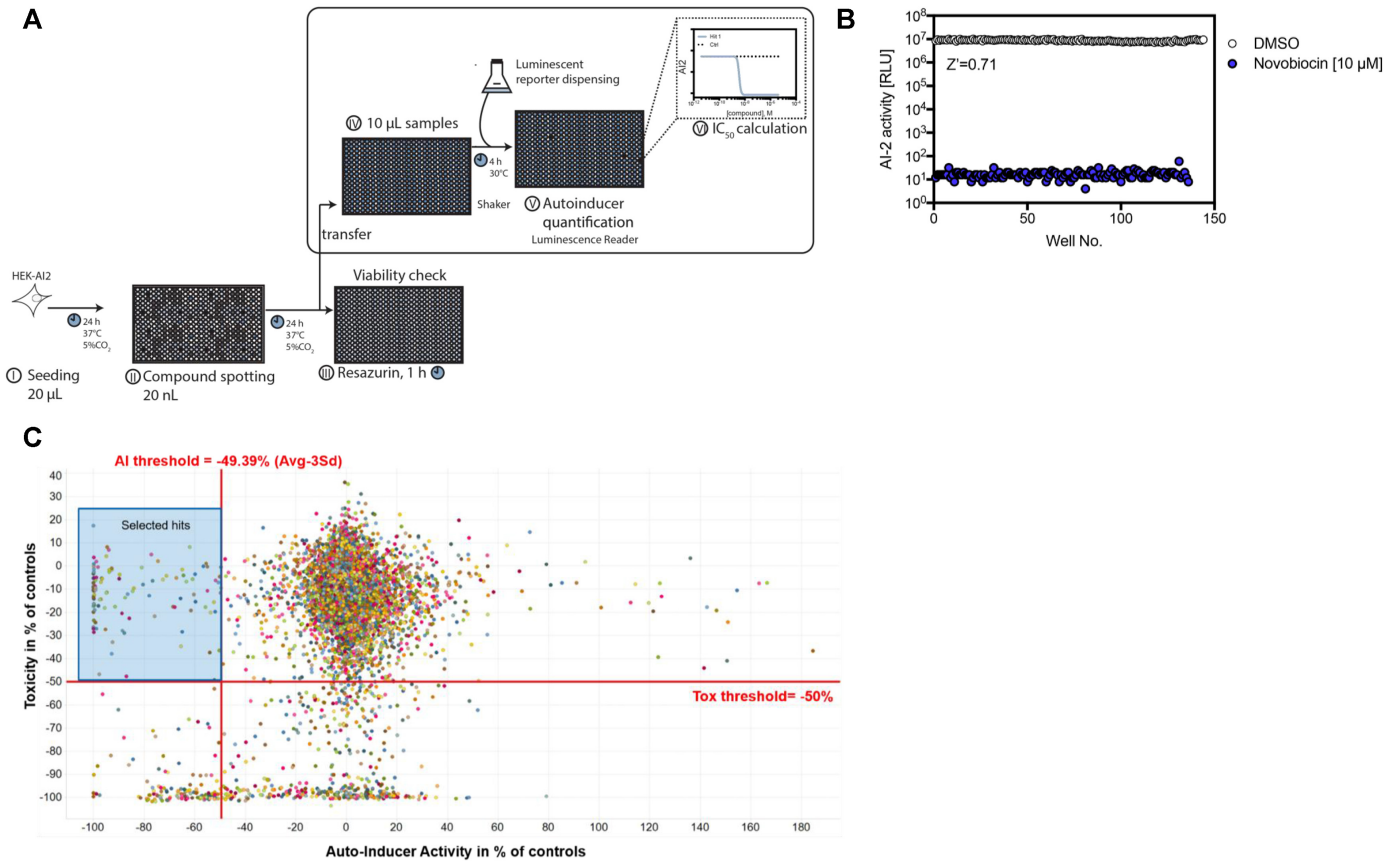
Cell-based primary screen for AI-2 biosynthesis inhibitors. (**A**) Detailed assay protocol of the primary screen using engineered mammalian cells to identify inhibitors of quorum sensing. HEK_AI2_ cells were seeded into 384-well plates and exposed to 20 nl of screening compounds in duplicate (5 μM final concentration), active control compound (novobiocin, 10 μM) and toxicity control compound (staurosporin, 20 μM) or DMSO as a negative control. Aliquots of 10 μl of culture supernatants were transferred to black-bottomed assay plates and mixed with 40 μl of *V. harveyi* MM32 (AI-2 biosensor). AI-2-induced bioluminescence was measured 4 h later and normalized against the controls. In a secondary screen, the dose-response relationships of hit compounds are analyzed. To simultaneously assess effects on cell viability, compound-treated cells were supplemented with PrestoBlue and incubated for 1 h prior to resorufin fluorescence quantification. (**B**) Z’-evaluation of the AI-2 inhibitor assay. HEK-293_AI2_ grown on 384-microwell plates were treated with control compounds (active control: novobiocin, 10 μM; negative control: DMSO) and incubated for 24 h. The AI-2-induced luminescence of the cell supernatants was quantified to determine the statistical effect size (Z’ = 0.71). (**C**) Discovery of AI-2 biosynthesis inhibitors by screening a library of publicly accessible compounds. The effects of 5,283 individual compounds on AI-2-induced bioluminescence and cell viability-dependent fluorescence were scored in parallel. Bioactivity and viability are calculated as percentages, based on the positive control value (taken as 100%) and negative control value (taken as 0%). The blue translucent background indicates the range of expected hit compounds. Data points in (C) represent means of biological duplicates.

We confirmed that AI-2 production and secretion by engineered HEK_AI-2_ cells could be precisely and reliably quantified in the culture supernatant by addition of the AI-2-sensitive reporter bacterium *V. harveyi* (MM32), which allows rapidly profiling of the quorum-sensing molecule by means of bioluminescence-based assay (34). The functional combination of HEK_AI-2_-mediated AI-2 production with *V. harveyi* (MM32)-based AI-2 quantification provides a potent mammalian cell-based assay platform for the detection of bioavailable, non-cytotoxic and target-specific small-molecular drugs quenching the production of AI-2 (Figure 2A). To set a benchmark for larger-scale screening, the cell-based AI-2 production assay was validated by using the *V. harveyi*-killing antibiotic novobiocin (35); this completely shut down the bioluminescence of *V. harveyi* (MM32) (Figure 2B). In parallel, the viability and metabolic integrity of HEK_AI-2_ were profiled by means of resazurin assay to detect test-compound-associated cytotoxicity (see Supplementary Figure S1).

### Discovery of quorum-quenching compounds in a chemical library

Capitalizing on the robust assays for both bioactivity (*Z*’ value of 0.71) and viability (*Z*’ value of 0.73), we upscaled our system for compatibility with an automated high-throughput industrial screening platform and tested a library of over five thousand commercially available compounds with validated mechanisms of action. Probing this chemical library in duplicate yielded 66 hit compounds (hit rate: 1.2%) that were bioavailable and showed quorum-quenching activities, while lacking substantial metabolic impact or cytotoxicity towards human target cells (Figure 2C). To mitigate off-target effects, only compounds decreasing AI-2 production by over 50% while maintaining human cell viability and metabolic integrity >50% were considered for a secondary screen of the dose-response relationship in human cells.

### Validation and potency quantification of quorum-quenching hit compounds

The AI-2-specific quorum-quenching activity of the hit compounds was confirmed by dose-dependence analysis using the HEK-293_AI2_-*V. harveyi* (MM32) AI-2 detection platform to determine the half-maximal inhibitory concentration (IC_50_) of the individual compounds. Among the quorum-quenching drug candidates from the primary screen, 30 compounds inhibited AI-2 production with IC_50_ values lower than 5 μM (Table 1), and among them, 11 compounds dose-dependently inhibited AI-2 production even in the submicromolar range (Supplementary Figure S2). Hit compounds that were listed in public databases as having antibiotic activity were excluded from consideration as they would decrease the bioluminescence simply by killing the reporter strain, *V. harveyi* (MM32) (Table 1). Fortunately, nine of the most potent AI-2 production-inhibiting compounds did not show any antibiotic activity. These included formycin A (36) and MT-DADMe-ImmA (37), which have previously been shown to exhibit quorum-quenching activity by inhibiting MTAN (Table 2). Rediscovery of established quorum-quenching compounds is a potent validation of this cell-based quorum-quenching drug-discovery platform.

**Table 1.** Hit list from screening. Validated compounds decreasing AI-2 activity are listed in order of efficacy (IC_50_). Expected antibiotic activity is based on PubChem annotations of biological activity

Hit no.	PubChem CID	Compound name	IC_50_ (μM)	Viability of Ctrl (%)	Antibiotic activity	Ref.
1	24849323	SCHEMBL12230405	0.15	–4.3	yes	
2	119182	Clofarabine	0.16	15.42	no	
3	656970	MT-DADMe-ImmA	0.17	6	no	(37)
4	10467650	CHEMBL283366	0.18	5.3	yes	
5	37542	Ribavirin	0.19	–1.95	no	(67)
6	18343	Doxifluridine	0.24	–0.0725	no	
7	3197	Levomycin	0.37	–19.76	yes	
8	3385	5-Fluorouracil	0.46	1.53	(yes)	(62)
9	24849362	SCHEMBL12230407	0.63	7.61	yes	
10	57937088	SCHEMBL12230409	0.64	–0.56	yes	
11	5790	Floxuridine	0.77	–22.28	no	
12	10475188	Actinonin	1	–11.93	no	(68)
13	9930332	SCHEMBL14510040	1.09	7.04	yes	
14	3255	Erythromycin Stearate	1.14	7.92	yes	
15	62860	Erythrohycin Gluceptate	1.31	9.37	yes	
16	54697674	Mocimycin	1.37	4.99	yes	
17	9918244	Erythromycin B	1.42	4.99	yes	
18	169674–35-5	NSN_RD-82-UC58	1.6	5.92	no	
19	12560	Erythromycin	1.93	3.21	yes	
20	13248213	CTK8J4091	2.04	5.73	no	
21	447199	Formycin	2.11	–20.11	(yes)	(36,69,70)
22	6857733	Triostin A	2.14	–37.96	yes	
23	6323490	Rifabutin	2.19	–5.07	yes	
24	329823987	Rifapentine	2.53	–0.98	yes	
25	3255	Erythromycin Stearate	2.59	–0.063	no	
26	54678491	Novobiocin derivate	3.33	3.63	yes	(71)
27	5479530	Ceftriaxone	3.49	–4.78	yes	
28	23418	Ormetoprim	3.66	–4.3	yes	(72)
29	441401	Linezolid	4.16	0.38	yes	
30	162715	Griseoviridin	4.55	4.96	yes	

**Table 2.** Annotation of hit compounds validated in secondary screen

Compound	Reported IC_50_ (*in vitro*)	Mechanism of action	Novelty
Clofarabine		Antineoplastic nucleoside Metabolic inhibitor (73)	yes
MT-DADMe-ImmA	*K*_i_ = 2 pM (74)	MTAN inhibition (74)	no
Ribavirin		Antiviral nucleoside Antimetabolite, antivirulence activity (67)	yes
Doxifluridine		Nucleoside analog prodrug (75)	yes
5-Fluorouracil		Nucleoside analog (75)	no (63,76)
Floxuridine		Nucleoside analog prodrug (75)	yes
CTK8J4091		Nucleoside analog (75)	yes
Formycin	IC_50_ = 57 μM/*K*_i_ = 10 μM (77)	Competitive MTAN inhibition (69,77)	yes (70)
Ormetoprim		DHFR inhibition (78)	no

### Target specificity of validated hit compounds

**Figure 3.**
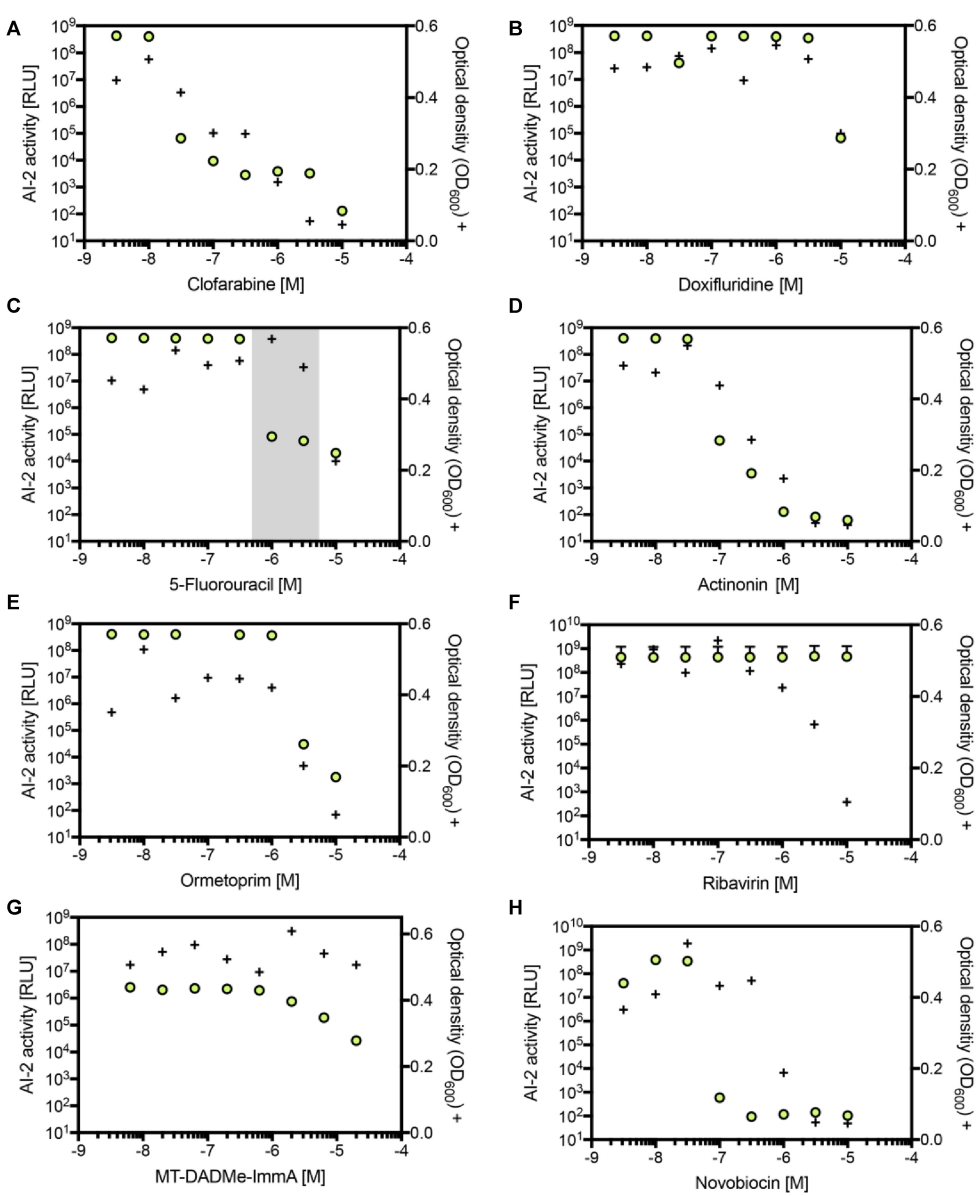
Bioactivity of hit candidates against *Vibrio harveyi*. Effect on bacterial growth. *V. harveyi* (BB170) was grown in the presence of individual compounds (**A–H**) for 5 h and the bioluminescence (green circles) was quantified. After a total of 24 h, bacterial growth (+) was evaluated by measuring the optical density (OD_600_). The gray translucent background in (**C**) represents the narrow range within which the compound reduces bioluminescence without affecting growth. Data show the means ± SD of three experiments.

Although the cell-based assay for the discovery of AI-2 biosynthesis inhibitors was designed to reveal compounds targeting the synthetic LuxS/MTAN methylation cycle bypass without affecting the metabolism of human cells, potential off-target effects impacting related metabolic pathways or bacterial growth still require careful examination. Therefore, we first examined the candidates’ interference with the growth of *V. harveyi* and *E. coli*. Indeed, many of the tested quorum-quenching drug candidates dose-dependently decreased the growth of *V. harveyi* (Figure 3A–H), and clofarabine showed the most potent effect (TC_50_ = 4.4 × 10^−7^) (Figure 3A). On the other hand, 5-FU, a licensed anti-cancer therapeutic that has not been reported to show quorum-quenching activity, reduced the bioluminescence without decreasing the growth of *V. harveyi* (Figure 3C), and also interfered with *E. coli* quorum sensing (Supplementary Figure S3a–d). 5-FU was therefore chosen for follow-up *in vivo* studies.

### 5-FU is a quorum quencher effective against MRSA in vitro and in vivo

Staphylococci, including *Staphylococcus aureus* (SA) and the coagulase-negative *Staphylococcus epidermidis* (SE), have evolved quorum-sensing systems that enable cell-to-cell communication. SE is a normally harmless commensal bacterium found on the skin, but under certain conditions, especially when a medical device is involved, it can become invasive and colonize the device. To validate the staphylococcal AI-2-specific quorum-quenching capacity of 5-FU, we first investigated its in vitro activity against SE 1457 wild type. 5-FU reduced AI-2 activity dose-dependently (Supplementary Figure S4a). To examine whether synthetic AI-2 supplementation would restore the phenotype impaired by 5-FU, the AI-2 precursor DPD was added directly to SE in combination with increasing 5-FU concentrations. In the presence of DPD, the AI-2 activity returned almost to baseline (Supplementary Figure S4b).

**Figure 4.**
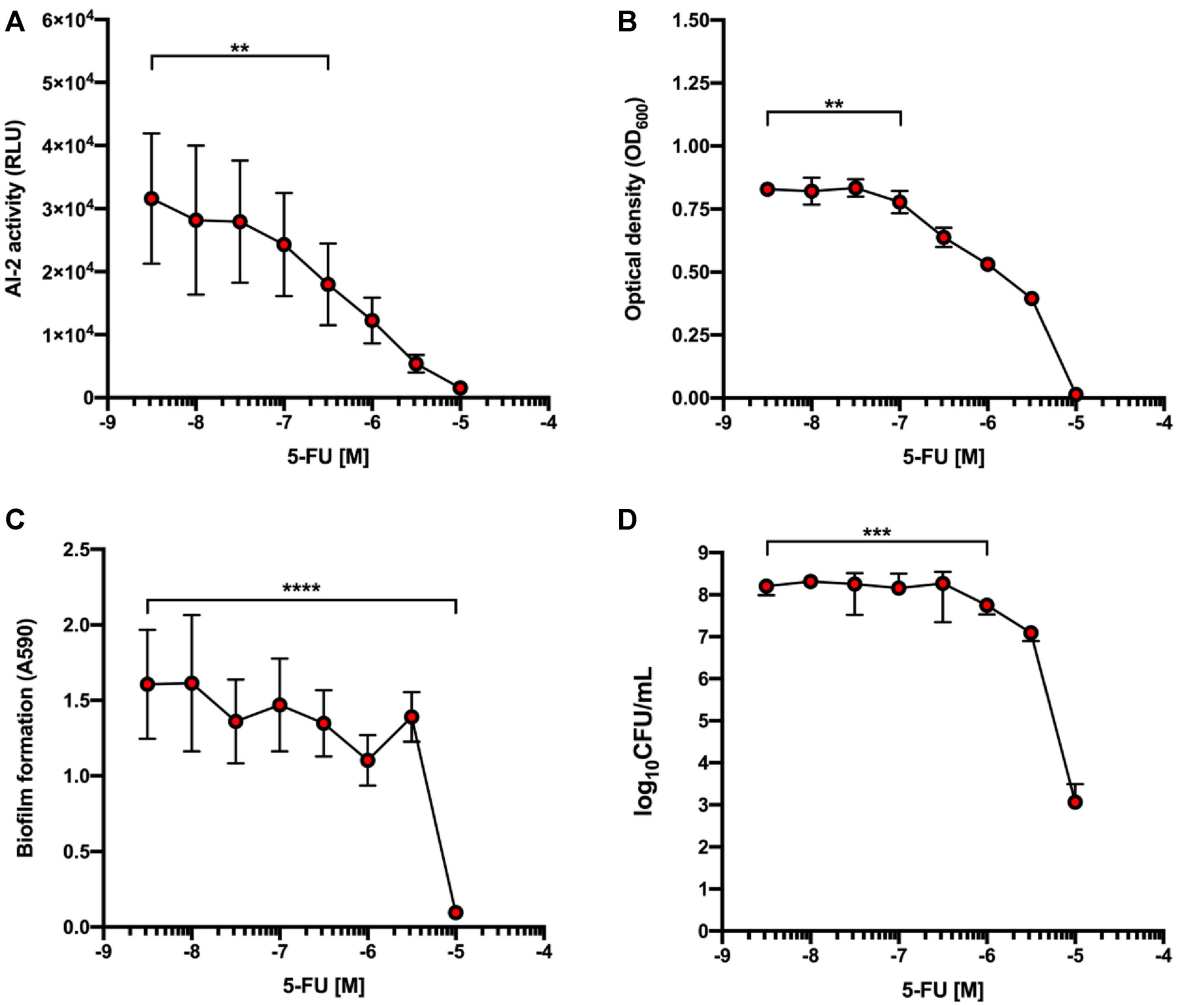
Bioactivity of 5-FU against MRSA ATCC 43300. (**A**) Quorum sensing-associated AI-2 activity of MRSA 43300 grown for 3 h with different 5-FU concentrations. (**B**) Effect of different 5-FU concentrations on MRSA 43300 growth. (**C**) Interference with biofilm formation. (**D**) Effect on adherent MRSA 43300. Data points represent three biological replicates expressed as mean ± SD (**P < 0.01; ***P < 0.001; ****P < 0.0001).

Even though SE exhibits considerably higher AI-2 activity than MRSA, the clinically relevant human pathogen MRSA (7,38) expresses more virulence factors that facilitate its spread and survival. To confirm the AI-2-specific quorum-quenching activity of 5-FU, we next examined its in vitro and in vivo activity against MRSA ATCC 43300. 5-FU dose-dependently reduced MRSA’s AI-2 production and quorum-sensing capacity (Figure 4A) and significantly decreased bacterial growth at the concentration of 0.1 μM (Figure 4B), which is an order of magnitude lower than the 5-FU concentration that is clinically relevant for anti-cancer therapy (39). At this concentration, 5-FU not only reduced the number of adherent MRSA but also significantly decreased biofilm formation (Figure 4C, D). To rule out possible artefacts caused by the vehicle (DMSO) used for 5-FU administration, the influence of DMSO was monitored in parallel. We observed that DMSO started to impact growth and biofilm formation at the highest concentration applied (corresponding to 10 μM 5-FU), though the effect did not reach statistical significance (data therefore not shown).

**Figure 5.**
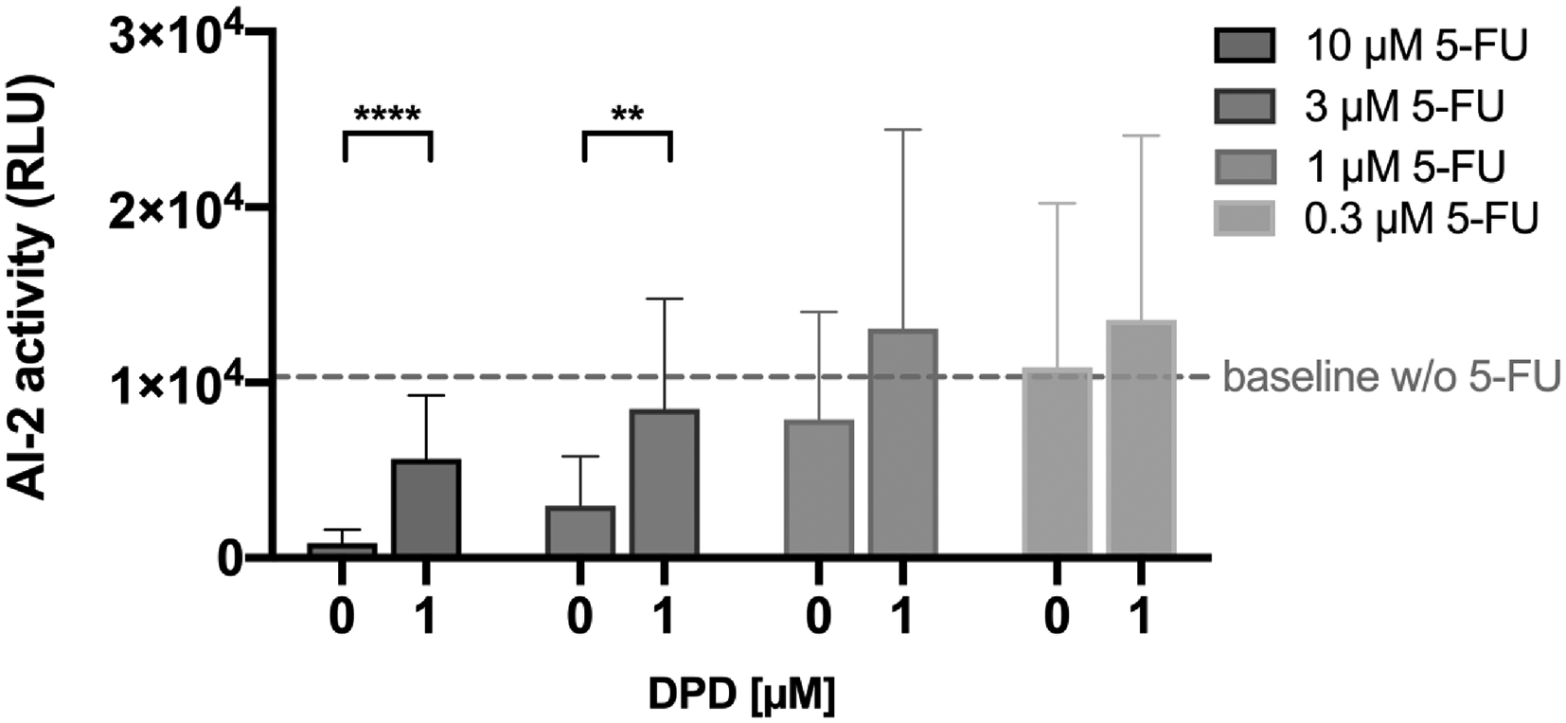
Phenotype restoration with synthetic AI-2 supplementation. AI-2 activity of MRSA ATCC 43300 was evaluated with and without 1 μM DPD in the presence of different 5-FU concentrations. The dashed line indicates the baseline level of AI-2 of MRSA ATCC 43300 in the absence of 5-FU. Values represent 3 biological replicates expressed as mean ± SD (**P < 0.01; ****P < 0.0001).

To examine whether the decrease of AI-2 activity caused by 5-FU is reversible, we supplemented the 5-FU-challenged MRSA with the AI-2 precursor DPD. The added DPD counteracted the effect of 5-FU, while showing little influence on the baseline AI-2 activity of MRSA 43300 (Figure 5). These findings support the idea that 5-FU slows down AI-2 biosynthesis rather than blocking AI-2 sensing.

**Figure 6.**
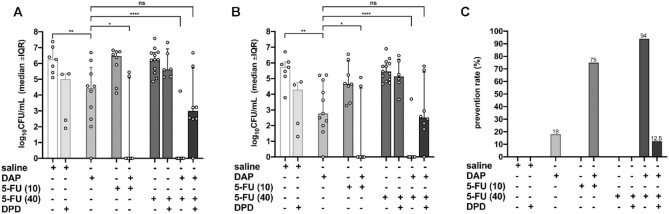
Prophylactic effect of 5-FU against MRSA ATCC 43300 in a murine tissue cage infection model. Read-outs were obtained two days after infection with 965 CFU/cage and treatment with saline, saline with 1 μM AI-2, 50 mg/kg daptomycin (DAP), 10 mg/kg 5-FU with or without 50 mg/kg DAP, 40 mg/kg 5-FU with or without 50 mg/kg DAP or 40 mg/kg 5-FU with 50 mg/kg DAP and 5.2844 mg/kg DPD, as indicated below the panels. (**A**) Effect on planktonic MRSA 43300 two days post-infection. (**B**) Effect on adherent MRSA after explantation and sonication. (**C**) Prevention rate. Values in (A) and (B) are median ± IQR and those in (C) are % (*P > 0.5; **P < 0.001; ****P < 0.0001; ns, not significant).

To assess 5-FU’s quorum-quenching anti-infective potential in vivo, we employed the foreign-body mouse infection model, which simulates human implant-associated infections (40). Mice were prophylactically treated with 5-FU (10 mg/kg or 40 mg/kg) in combination with or without daptomycin (DAP). All drug concentrations were within the human therapeutic dosage range (41). In order to assess if the effect of 5-FU is mediated by inhibition of AI-2 production, we simultaneously administered synthetic AI-2 (DPD). While treatment with 5-FU or DAP alone failed to clear planktonic MRSA infections (Figure 6A), simultaneous prophylactic treatment of animals with 5-FU and DAP prevented MRSA infection and cleared the pathogen (Figure 6A). Likewise, prophylactic administration of 5-FU and DAP prevented biofilm formation and completely eradicated MRSA, though neither 5-FU nor DAP alone was sufficient to contain the infection (Figure 6B, C). Interestingly, the presence of DPD reversed the 5-FU-dependent inhibition of planktonic (Figure 6A) and adherent bacteria (Figure 6B) when co-administered with DAP. Similarly, DPD decreased the prevention rate to the level observed for DAP monotherapy (Figure 6C).

## DISCUSSION

Overcoming antibiotics resistance is one of the most critical, complex and pressing healthcare challenges of the 21st century. The rapid development of resistance, as well as the poor profitability of these life-saving drugs, which often lose efficacy even before coming off patent, is making more-of-the-same development of novel antibiotics or new antibiotic derivatives scientifically questionable and economically non-viable (38,42,43). Therefore, new anti-infective strategies, as well as novel blueprints for small-molecular drug discovery, are urgently needed to cope with the globally increasing prevalence of multidrug-resistant pathogenic bacteria (17,44). Several proof-of-concept studies have appeared, suggesting the use of recombinant phages to target bacteria (45), small-molecular drugs to switch off antibiotic resistance genes in *Mycobacterium tuberculosis* (22,23), antibiotic adjuvants to increase efficacy (46), or immuno-mimetic designer cells to detect and kill multidrug-resistant *S. aureus* (40). In particular, studies on quorum-quenching drugs that interfere with the pathogens’ inter-species and intra-population molecular communication to coordinate persistence (47), virulence (18) and biofilm formation (10,48) are gathering momentum, because non-killing drugs that interfere with quorum-sensing should impose low selection pressure, and may eliminate, reduce or delay the emergence of resistance. Non-limiting examples of quorum-quenchers include brominated furanones (49), autoinducer-degrading enzymes (50) and the established antibiotic azithromycin (51). In addition, coatings functionalized with 5-FU have shown promising results in phase-1 clinical trials for the treatment or prevention of implant-associated infections (52); however, the molecular target(s) of 5-FU’s anti-infective activity has remained elusive until now. Despite the great prospects for new quorum-quenching drugs, every new class of drugs may have side effects or show off-target activities. Therefore, expanding the therapeutic space of licensed drugs with a proven track record of tolerability by finding alternative targets and activities may be a rapid and efficient strategy for bridging the gap until novel approaches can be brought into clinical use.

Drug discovery has not seen much conceptual progress in recent decades. Structure-function predictions, drug-target fitting and high-throughput screening have become increasingly sophisticated, but hit-to-lead development has remained challenging due to limited bioavailability, poor pharmacokinetics and/or cytotoxicity of many drug candidates (53). Thus, although cell-based assays are more expensive to set-up and operate, they may offer advantages over classic in-vitro drug screening, in that they can validate the function, cytotoxicity, bioactivity and bioavailability of drug candidates in an all-in-one test format. Non-limiting proof-of-principle examples of cell-based screening assays include the discovery of novel anti-infective activities (20,21), peptides ameliorating chronic pain (54), and drug candidates that switch-off antibiotic resistance genes (23). Indeed, a drug switching off the antibiotic resistance of *Mycobacterium tuberculosis*, a century-old plague, is currently under phase I clinical trial by a Bioversys-GlaxoSmithKline consortium (23). These small-molecular drugs are based on early hits in cell-based assays revealing compounds that switch off the pathogen's intrinsic resistance to the last-line antibiotic ethionamide (22). In contrast to classical antibiotics which eliminate target pathogens, thereby creating a strong selective pressure that drives the emergence of resistance, there is hope that antibiotics co-administered with compounds that switch off antibiotic-resistance may reduce the selective pressure and thus delay the onset of resistance. But, as this approach still focuses on actively killing the pathogen, resistance is still expected to arise eventually, as in classical antibiotic therapies. Consequently, it is conceivable that attenuation, rather than killing, of the pathogen may become a valid strategy to avoid the selection of resistant populations in future anti-infective therapies. Following Darwinian principles, attenuation of pathogenic traits to increase fitness is typically observed in the co-evolution of host-pathogen interactions. Therefore, non-killing interventions leading to attenuation of host-pathogen interactions may open up new opportunities for anti-infective therapies.

Studies of host-pathogen interactions at the molecular level are frequently done in co-culture systems of bacterial pathogens and host cells (31,55). Such co-cultures not only provide new insight into host-pathogen crosstalk, but also at the same time provide a framework for cellular assays for the discovery of drugs interfering with the host-pathogen interaction. However, the application of synthetic biology principles to engineer mammalian cells with functionalized bacterial circuits and user-defined drug targets has largely eliminated the need for host-pathogen co-cultivation, and instead has enabled the development of simple cell-based assays, thereby increasing the repertoire of drug-screening tools (12,56). For example, pioneering transfer of pathogen-derived repressor-operator gene switches into mammalian cells enabled hit discovery of novel streptogramin antibiotics (21) and anti-microbacterial drugs (22,57). Using the same principle for engineering drug-target-specific assay cell lines (22,58–60), but replacing the reporter gene with a therapeutic effector gene, has resulted in novel cell-based anti-infective therapies (40,55). For example, implant-associated infections by *S. aureus* could be prevented as well as cleared by using engineered human-cell implants that detected the presence of the pathogen and coordinated a rapid, reversible and dose-dependent peptide expression response to eliminate biofilm as well as planktonic multidrug-resistant MRSA (40). Overall, designer cell lines incorporating critical pathogen-derived circuits can serve as (i) cell-culture based systems to reveal molecular information on host-pathogen interactions, (ii) cell-based therapies to prevent and cure bacterial infections and (iii) cell-based assays to screen novel anti-infective drugs. Running such cell-based assays in an academic-industrial context using libraries of compounds with established activities is a successful strategy for repurposing licensed compounds with a therapeutic track record, enabling rapid clinical application, and so is particularly relevant for last-line anti-infective therapies. Indeed, this approach identified the licensed chemotherapeutic pyrimidine analogue 5-fluorouracil, one of the WHO essential medicine for chemotherapy (61), as a potent antimicrobial drug. But, although empirical data show that 5-FU has antibiotic-boosting activity, the molecular mechanism has remained a mystery for decades (62,63,64–66), and consequently, a rational basis for its therapeutic application as an anti-infective has been lacking. Now, we have shown that 5-FU quenches the quorum-sensing activity in an *in vivo* model of implant-associated MRSA infections to such an extent that the infections could be completely cleared by the last-line antibiotic daptomycin, which achieves <20% clearance rate when used alone (26). Notably, the addition of the AI-2 precursor DPD reinstated this low rate of clearance and prevention by neutralizing the 5-FU-dependent quorum quenching.

The cell line used for initial screening incorporated *E. coli*-derived AI-2 biosynthetic enzymes that share sequence identities of 35% for LuxS and 53% for MTAN with the *S. aureus* homologues. However, AI-2 interference of identified hit compounds was subsequently confirmed in diverse bacterial species, so we believe this is not problematic. Furthermore, the anti-infective effect of 5-FU originally discovered in engineered human cells was also observed in SE, which exhibits pronounced AI-2-mediated quorum sensing and stronger biofilm formation than MRSA (26). Quorum sensing is inherently linked to biofilm formation, so it is not unexpected that both traits respond to 5-FU in a similar manner.

Although it is still too early to speculate about any common molecular basis for simultaneous interference with bacterial and tumor growth, the observation that a simple pyrimidine can block both DNA synthesis and quorum-sensor biosynthesis may represent a valuable clue for the development of future anti-infective strategies. Indeed, we propose that the anti-neoplastic drug 5-FU could as of now be considered for anti-infective therapy of implant-associated MRSA infections.

## DATA AVAILABILITY

All data and materials are available upon request. Sequences of key expression vectors have been deposited in GenBank: pFS83, MF688636; pFS84, MF688635.

## Supplementary Material

gkab251_Supplemental_File
