# Supplementary material for: 5-Fluorouracil blocks quorum-sensing of biofilm-embedded methicillin-resistant Staphylococcus aureus in mice
Source: Nucleic Acids Res. 2021 Apr 15;49(13):e73. doi: 10.1093/nar/gkab251 (PMC8287944; doi:10.1093/nar/gkab251)
Supplement: gkab251_Supplemental_File [file gkab251_supplemental_file.docx]

#### Supplementary Figures

**Supplementary Figure 1 | Resazurin Viability Assay.** HEK_AI-2_ cells were treated with active toxic control (staurosporin) or DMSO as a negative control, and cell viability was quantified using fluorescent resazurin as an indicator of respiratory activity.

**Supplementary Figure 2 | Validation screen and IC_50_ determination.** Dose-response relationship of hit compound-treated AI-2 producer cells. Cells were exposed to increasing concentrations of selected purified compounds (a-x) for 24 h. AI-2 activities in the culture supernatants were measured, normalized against (in-)active controls, and fitted using a 4-parameter logistic regression. Data points represent 3 biological replicates.


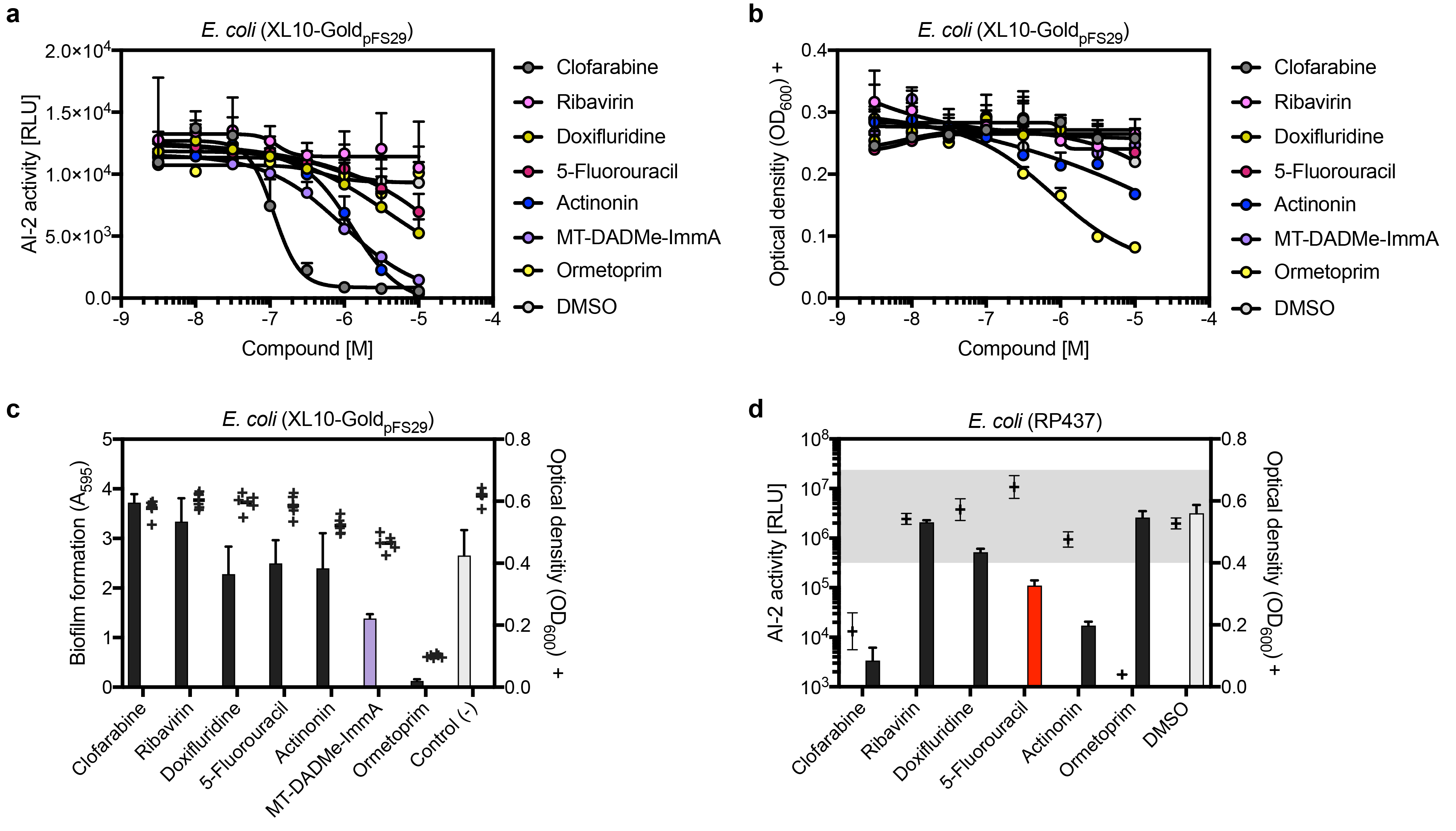


**Supplementary Figure 3 | Effect of hit candidates on *Escherichia coli*.** (a) Modulation of AI-2 production. AI-2-secreting *E. coli* (XL10-Gold) was grown for 3 h and AI-2 activity in the supernatant was quantified. (b) Effect on growth*.* Optical density (OD_600_) was measured after 24 h as a measure of cell growth. (c) Interference with biofilm formation. *E. coli* (XL10-Gold) was grown for 24 h in the presence of test compounds (10 µM). Then, planktonic cell growth (OD_600_) was measured and biofilm formation was quantified by means of crystal violet staining. (d) AI-2-secreting *E. coli* (RP437) was grown for 3 h in the presence of different compounds (10 µM), and then AI-2 activity in the supernatant was quantified. Optical density (OD_600_) was measured after 24 h.

**Supplementary Figure 4 | Bioactivity of 5-FU against *Staphylococcus epidermidis* (SE).** a) Quorum-sensing-associated AI-2 activity of SE 1457 grown for 3 h in the presence of different 5-FU concentrations (note log scale). b) Reversing effect of 1 µM DPD on the AI-2-reducing action of 5-FU (dashed line indicates AI-2 baseline of SE 1457 without 5-FU). Values represent 3 biological replicates expressed as mean ± SD (*, p < 0.05; **, p < 0.01).
